# Decoding free-throw accuracy: a 2D biomechanical comparison of shooting mechanics in athletes and novice basketball players

**DOI:** 10.3389/fspor.2026.1834844

**Published:** 2026-06-10

**Authors:** Peng Wang, Yali Xu, Yunjie Li, Changwei Chen, Rahmat Hidayat

**Affiliations:** Department of Fundamental Education, Qilu Institute of Technology, Shandong, Jinan, China

**Keywords:** 2D kinematics, basketball, elbow extension, free-throw, release height, shooting accuracy

## Abstract

This study examined the key biomechanical determinants of free-throw accuracy by comparing 2D kinematic characteristics between trained basketball athletes and novice players. Fifty right-hand dominant participants (25 athletes, 25 novices; age 18–24 years) performed 20 standardized free-throw trials after familiarization. Upper and lower-limb joint angles, trunk lean, hand velocity, and ball-release parameters (height, angle, velocity) were extracted from sagittal-plane videos using Dartfish software, with high intra- and inter-rater reliability (ICC = 0.88–0.95). Successful and unsuccessful shots were analyzed separately. Statistical comparisons used independent *t*-tests or Mann–Whitney *U*-tests with Bonferroni correction, and effect sizes (Cohen's d) were calculated. Correlations between kinematic variables and shot success were examined for each group. Athletes had superior anthropometrics, physical performance, and higher absolute kinematic values than novices. Successful shots in both groups featured greater elbow extension (Athletes: 158.1 ± 3.1° vs. 152.3 ± 3.8°; Novices: 150.5 ± 4.0° vs. 145.0 ± 4.4°) and higher ball release height (Athletes: 2.28 ± 0.06 m vs. 2.19 ± 0.07 m; Novices: 2.15 ± 0.08 m vs. 2.05 ± 0.09 m), which showed the strongest correlations with success (r ≥ 0.63, *p* < 0.01). Shoulder, wrist flexion and hand velocity contributed moderately, whereas lower-limb and trunk kinematics mainly supported stability and energy transfer. Directional patterns for successful shots were consistent across skill levels, though athletes exhibited higher absolute values and reduced variability. Free-throw accuracy is primarily determined by distal segment mechanics, particularly elbow extension and release height, supported by coordinated shoulder elbow wrist sequencing. These findings provide evidence-based guidance for coaching interventions emphasizing release mechanics over lower-limb force production to enhance shooting performance across skill levels.

## Introduction

Free-throw shooting is a fundamental skill in basketball and a critical determinant of game outcomes, particularly during decisive moments characterized by high psychological pressure (1–4). Unlike open-play shooting, free throws are executed in a relatively controlled and static environment, minimizing external interference (5, 6). Consequently, free-throw success depends predominantly on technical consistency, motor control, and mechanical efficiency of movement, making it an ideal task for biomechanical investigation (7–9).

From a biomechanical perspective, free-throw accuracy is governed by the coordinated interaction of lower- and upper-limb kinematics, proximal-to-distal sequencing, and the precise regulation of joint angles and release parameters at ball release (10–12). Previous studies have demonstrated that skilled basketball players typically exhibit more stable movement patterns, reduced kinematic variability, and superior inter-segmental coordination compared with less experienced players (13–15). However, the specific biomechanical mechanisms that differentiate free-throw shooting accuracy between trained athletes and novice players remain insufficiently elucidated.

Existing research on basketball free-throw performance has largely focused on outcome-based measures, such as shooting percentage or success rate, providing limited insight into the underlying movement mechanics responsible for performance differences (7, 16, 17). Moreover, although three-dimensional (3D) motion analysis is often regarded as the gold standard in biomechanical research, its high cost, technical complexity, and limited accessibility restrict its application in practical coaching and training environments. In contrast, two-dimensional (2D) biomechanical analysis offers a more feasible, cost-effective, and ecologically valid alternative for evaluating shooting mechanics (18–20). Importantly, given that free-throw shooting is predominantly executed in the sagittal plane, 2D analysis may be sufficient to capture key kinematic variables associated with shooting performance (21, 22). Despite this potential, 2D approaches remain underutilized in comparative studies examining skill-level differences in free-throw shooting, highlighting the need for further investigation in applied settings.

Furthermore, much of the current literature has concentrated on elite or highly trained basketball players, resulting in a limited understanding of how shooting mechanics evolve across different levels of expertise (23–25). Comparative investigations between athletes and novice players are essential for identifying skill-dependent biomechanical characteristics, which can inform evidence-based coaching strategies and facilitate the development of effective training interventions. Without such comparative analyses, it remains challenging to distinguish between movement patterns that are merely habitual and those that are mechanically optimal for achieving high shooting accuracy.

Therefore, there is a clear need for a comprehensive biomechanical examination that decodes free-throw accuracy by directly comparing shooting mechanics between athletes and novice basketball players using a 2D kinematic approach. Such an investigation can elucidate key mechanical variables associated with accurate shooting and clarify how movement coordination differs as a function of expertise. Accordingly, the purpose of this study was to analyze and compare the 2D biomechanical characteristics of free-throw shooting mechanics between trained basketball athletes and novice players. By identifying critical kinematic parameters that distinguish skilled from unskilled performance, this study aims to contribute to the scientific understanding of basketball shooting mechanics while providing practical insights for coaching, skill acquisition, and performance enhancement.

## Materials and methods

### Study design

This study employed a cross-sectional comparative observational design with a quantitative approach to examine differences in 2D biomechanical characteristics of free-throw shooting between trained basketball athletes and novice players. The independent variable was skill level (athlete vs. novice), while the dependent variables included 2D kinematic parameters, such as joint angles of the lower and upper limbs and ball-release parameters.All testing procedures were conducted on a basketball court to ensure measurement consistency and minimize external variability. The study was approved by the institutional ethics committee, and all participants provided written informed consent prior to data collection.

### Participants

A total of 50 participants aged 18–24 years were recruited using purposive sampling based on inclusion criteria, and divided into 25 athletes and 25 novices. The athletes were competitive basketball players with ≥5 years of structured training, regularly participating in regional or national competitions, and demonstrating a free-throw accuracy of ≥65% during preliminary screening. The novices were university students with no formal training or competitive experience in basketball, demonstrating a free-throw accuracy of ≤40%. All participants were right-hand dominant, free from musculoskeletal injuries in the past six months, and had normal or corrected-to-normal vision. Exclusion criteria included a history of upper or lower limb injury, medical or neurological conditions affecting shooting ability, use of medication affecting motor coordination, and inability to complete all testing procedures. An *a priori* power analysis using G*Power (α = 0.05; power = 0.80; effect size = 0.80) indicated that 25 participants per group were sufficient to detect significant differences between groups.

### Anthropometrics and physical performance

Participants’ height, weight, and body mass index (BMI) were measured using a stadiometer and calibrated scale (26). Agility was assessed via a 10-m shuttle run (six shuttles, total 60 m), recording the fastest of two trials (27, 28). Lower-limb power was measured with a manual vertical jump using the wall reach method, calculating the difference between standing reach and maximal jump height (29, 30). Muscular endurance was evaluated with 60-second push-up and sit-up tests, counting correctly executed repetitions (31, 32). Posterior chain flexibility was assessed with the sit-and-reach test, using the best of two trials (33, 34). All tests were performed under standardized conditions and supervision to ensure proper technique, providing data to interpret kinematic and biomechanical differences between trained athletes and novices.

### Experimental procedure

Prior to data collection, all participants underwent a standardized warm-up, including 5 min of light jogging and dynamic stretching of the upper and lower extremities. Participants then performed 10 submaximal free-throw attempts as a familiarization phase to stabilize shooting mechanics and become accustomed to the data collection procedures. Following familiarization, each participant performed 20 free-throw trials from the standard distance (4.57 m), with 30 s rest intervals between trials to minimize fatigue effects without disrupting shooting rhythm (8, 35). The number of trials was selected to provide sufficient repetitions for capturing consistent kinematic patterns while avoiding fatigue-induced alterations in shooting mechanics, which have been shown to influence movement characteristics in basketball shooting (3, 36). Moreover, previous biomechanical studies have employed a similar or smaller number of trials when analyzing shooting kinematics (6, 37), as the primary objective is to examine movement patterns rather than long-term performance variability. All attempts were recorded as successful (made) or unsuccessful (missed). Kinematic analysis was conducted separately for successful and unsuccessful shots, allowing comparison of the movement patterns underlying shot success and failure. This approach ensures that the data capture the full variation in shooting mechanics among participants and enhances the validity of the findings.

### Camera setup

**Figure 1:**
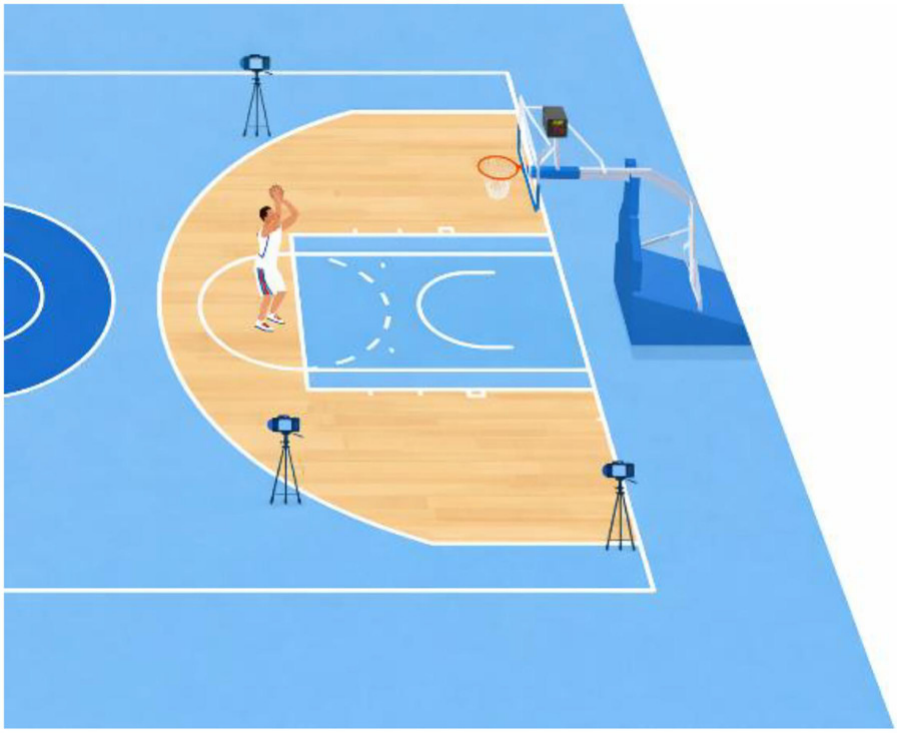
Illustrates the camera layout employed during the motion analysis.

Three high-speed digital cameras were used to record all free-throw trials from complementary perspectives. Two cameras were positioned on the right and left sides of the participants to capture bilateral sagittal-plane motion, while a third camera was placed at the corner of the court facing the basket to document ball trajectory and shot outcome (made or missed). The sagittal cameras were located 6 m from the free-throw line at a height of 1.5 m, with their optical axes oriented perpendicular to the primary plane of motion to minimize perspective distortion and parallax errors in two-dimensional recording (6, 38). All cameras were mounted on stable tripods and leveled horizontally to ensure consistent and stable imaging throughout data collection. Video recordings were captured at 240 frames per second (fps) to maintain temporal accuracy during critical movement phases, particularly at ball release. Prior to recording, camera position, focus, and field of view were verified to ensure visual clarity and spatial consistency across trials. In addition, a calibration procedure was performed using a reference object of known dimensions positioned within the sagittal plane of motion to enable conversion of pixel coordinates into real-world measurements for two-dimensional kinematic reconstruction (39, 40). The calibration object was aligned with the plane of movement to ensure accurate spatial scaling and to minimize perspective and parallax-related measurement errors (41, 42). This approach is based on the planar motion assumption commonly applied in two-dimensional biomechanical analysis. A visual representation of the camera setup used to capture the basketball free-throw motion is provided in Figure 1.

### Kinematic variables

Kinematic variables were divided into three categories: upper body, lower body, and ball-related variables (43–45). Upper body variables included shoulder, elbow, and wrist flexion/extension angles, trunk lean, and hand segment velocity, measured in the sagittal plane at key points such as maximal arm raise and ball release. Lower body variables comprised hip, knee, and ankle flexion/extension angles, as well as vertical displacement of the center of mass, capturing the contribution of the lower limbs to shooting stability and power generation. Ball-related variables included ball release height, release angle, and release velocity, calculated from sagittal camera footage, while shot outcome (made or missed) was verified using the corner camera. High-speed recording at 240 frames per second enabled accurate capture of rapid movement phases, particularly wrist motion and ball release. All angular measurements were expressed in degrees, velocities in meters per second, and heights in meters, providing standardized and quantifiable assessment of free-throw mechanics.

### Motion analysis

Recorded video data were analyzed using Dartfish motion analysis software. The software enabled frame-by-frame tracking of body segments and ball trajectories to extract key kinematic variables. Cameras were calibrated using a 1.00 m × 1.00 m grid with 10 cm divisions as the spatial reference (46, 47), allowing Dartfish to automatically compute a pixel-to-meter conversion factor of approximately 0.0025 m/pixel. Calibration accuracy was verified through Dartfish's internal validation, with the RMS error maintained below 1% of the calibration frame, demonstrating high precision in spatial reconstruction (48–50). This approach provided reliable, reproducible, and valid data for subsequent biomechanical analyses.

### The anatomical landmarks

**Figure 2:**
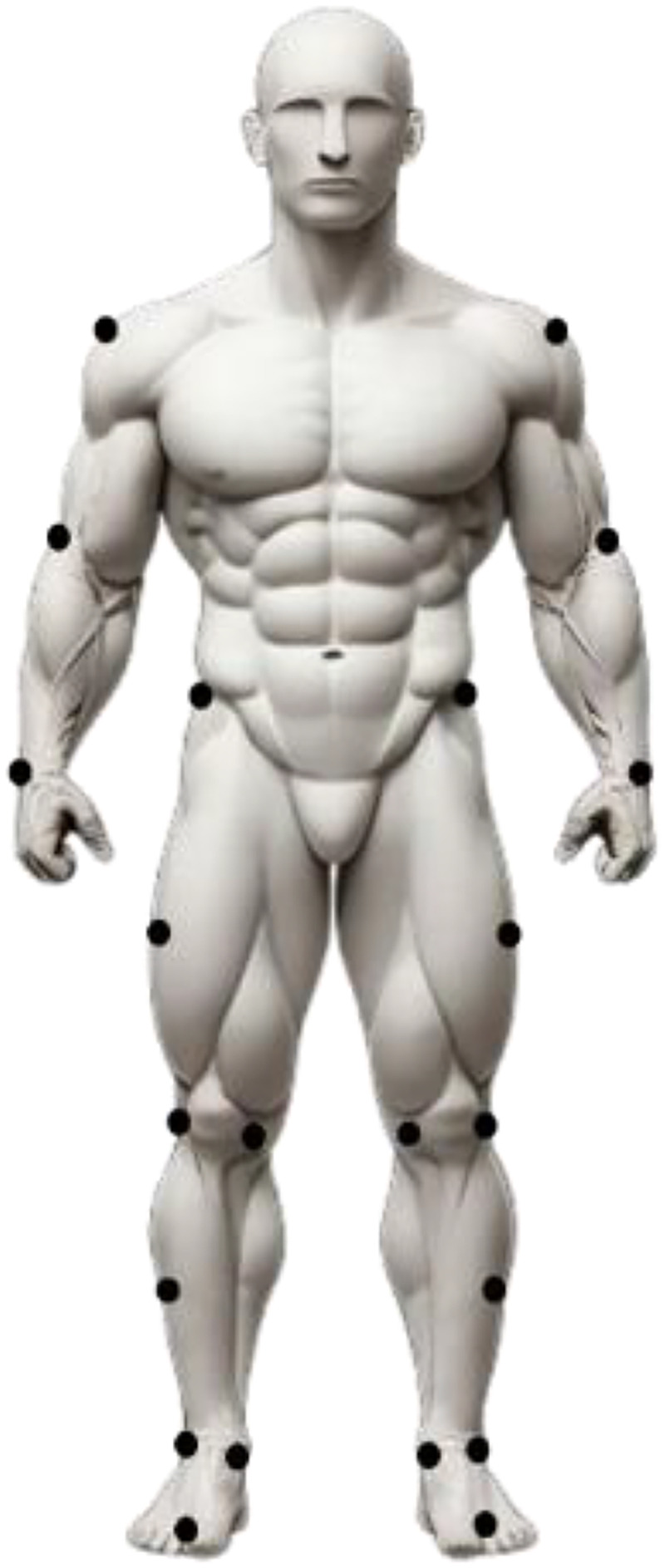
Marker placement on anatomical landmarks for kinematic analysis of the basketball free-throw.

Anatomical landmarks were manually digitized in the sagittal plane on a frame-by-frame basis using Dartfish motion analysis software (51). The selected landmarks acromion, lateral humeral epicondyle, radial styloid, greater trochanter, lateral femoral epicondyle, and lateral malleolus were chosen due to their clear visual definition and relevance for calculating segmental orientations and joint kinematics during the shooting task. All digitization procedures were performed by trained raters, with consistent procedures applied across all trials, following standardized anatomical definitions to ensure reliable landmark identification (52–54). To quantify digitization consistency, intra- and inter-rater reliability were evaluated using intraclass correlation coefficients based on absolute agreement models. The results demonstrated high reliability across all markers, with intra-rater ICC values ranging from 0.90 to 0.95 and inter-rater ICC values from 0.88 to 0.93. In accordance with established biomechanical reliability thresholds (55), these values indicate good to excellent agreement, supporting the methodological validity of the manual marker digitization approach. Despite these precautions, it is acknowledged that manual digitization of anatomical landmarks may introduce minor measurement errors that could influence joint angle estimation due to the subjective identification of joint centers and limitations in video resolution, which is a recognized limitation in two-dimensional biomechanical analysis (56–58). A visual representation of marker placement is provided in Figure 2.

### Statistical analysis

All statistical analyses were conducted using SPSS version 28.0 (IBM Corp., Armonk, NY, USA). Descriptive statistics, including mean ± standard deviation (SD), were calculated for all kinematic variables. Normality of data distribution was assessed using the Shapiro–Wilk test, and homogeneity of variance was tested with Levene's test. Based on these results, normally distributed variables were compared between athletes and novice players using independent-samples *t*-tests, while non-normally distributed variables were analyzed using the Mann–Whitney *U*-test. To account for multiple comparisons across all kinematic variables, the Bonferroni correction was applied. In addition, the Holm–Bonferroni sequential correction procedure was applied as a less conservative alternative to adjust for multiple comparisons while maintaining statistical power. Effect sizes were calculated using Cohen's d, interpreted as small (0.2), medium (0.5), and large (0.8) effects. Pearson correlation coefficients were computed for normally distributed variables, and Spearman correlation coefficients for non-normally distributed variables, to examine relationships between key kinematic parameters (e.g., release height, elbow angle, release velocity). Shot success was coded as 1 for made and 0 for missed trials, and correlations were calculated separately for Athletes and Novices to account for differences in skill level. All analyses were conducted separately for successful and unsuccessful free-throw trials to account for potential mechanical differences associated with shot outcome, with multicollinearity among key kinematic variables assessed using variance inflation factor (VIF) analysis. Furthermore, analysis of covariance (ANCOVA) was conducted to examine group differences while controlling for potential confounding effects of anthropometric variables, particularly body height. Adjusted means were calculated to determine whether differences in key kinematic variables, such as release height, remained significant after accounting for these covariates. Statistical significance was set at *p* < 0.05.

## Results

All participants completed the anthropometric and physical performance assessments, and the results are summarized in Table 1. Shooting performance differed significantly between groups. Athletes achieved a mean free-throw accuracy of 67.8 ± 6.5%, whereas novices demonstrated a substantially lower accuracy of 38.9 ± 7.2% (*p* < 0.001), indicating a clear difference in shooting performance between groups. No significant differences were observed between athletes and novices in age or body mass index (BMI). In contrast, athletes demonstrated significantly greater body height and body mass compared with novices (*p* < 0.05). Significant group differences were identified across all physical performance measures. Athletes outperformed novices in agility, completing the 10-m shuttle run in significantly shorter times (*p* < 0.001). Lower-limb power, assessed via vertical jump height, was also significantly higher in athletes than in novices (*p* < 0.001). Muscular endurance differed significantly between groups, with athletes performing a greater number of push-ups and sit-ups within the 60 s testing period (both *p* < 0.001). Additionally, athletes exhibited significantly greater posterior chain flexibility compared to novices, as indicated by sit-and-reach performance (*p* = 0.001).

**Table 1:** Anthropometric and physical performance tests (mean ± SD).

Variable	Athletes (*n* = 25)	Novices (*n* = 25)	*p*-value
Age (years)	21.2 ± 1.6	20.9 ± 1.7	0.48
Height (cm)	178.6 ± 6.4	172.3 ± 5.9	<0.01
Weight (kg)	72.8 ± 7.2	68.1 ± 6.8	0.02
BMI (kg·m⁻²)	22.8 ± 1.9	22.9 ± 2.1	0.86
Agility—10 m Shuttle Run (s)	14.21 ± 0.63	15.67 ± 0.78	<0.001
Vertical Jump Height (cm)	54.6 ± 6.8	41.3 ± 5.9	<0.001
Push-ups (reps/60 s)	42.5 ± 6.4	29.8 ± 5.7	<0.001
Sit-ups (reps/60 s)	48.9 ± 7.1	34.6 ± 6.3	<0.001
Sit-and-Reach (cm)	27.4 ± 5.2	21.6 ± 4.8	0.001

**Figure 3:**
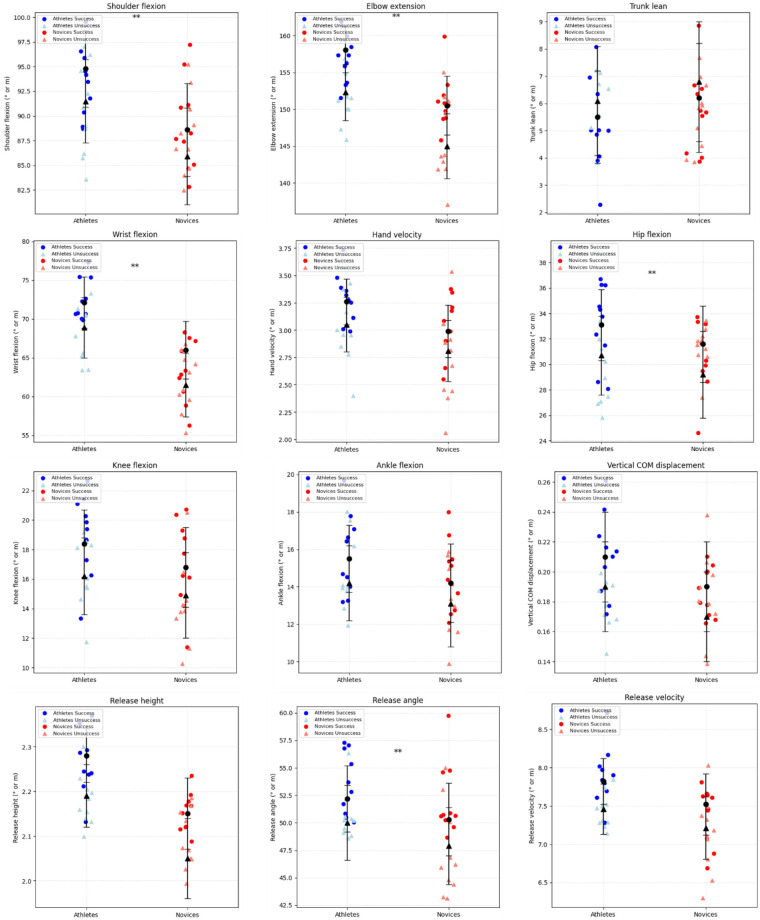
Comparison of kinematic and release variables between athletes and novices during the basketball free throw task. Scatter plots show individual data points for successful and unsuccessful free throw trials, with black markers representing group means ± SD. Significant between-group differences are indicated by ** (*p* < 0.01).

All statistical analyses were conducted separately for successful and unsuccessful free-throw trials. Variables meeting normality and homogeneity of variance assumptions were analysed using independent-samples *t*-tests, while non-normal variables were analysed using the Mann–Whitney *U* test. Bonferroni correction was applied to adjust for multiple comparisons across kinematic variables. Complete descriptive data are presented in Table 2. Figure 3 shows individual kinematic and release variables for athletes and novices during the free-throw task. Elbow extension angle differed significantly between successful and unsuccessful shots within both groups. In athletes, successful trials had higher elbow extension (158.1 ± 3.1°) than unsuccessful trials (152.3 ± 3.8°, *p* < 0.01, *d* = 1.92). A similar pattern was observed in novices (150.5 ± 4.0° vs. 145.0 ± 4.4°, *p* < 0.01, *d* = 1.92). Shoulder flexion and wrist flexion angles also showed significant differences between successful and unsuccessful trials within both groups, with moderate to large effect sizes. Hand velocity at ball release was greater in successful trials compared with unsuccessful trials in both athletes (*3.26* *±* *0.21* *m/s* vs. *3.05* *±* *0.25* *m/s*, *p* = 0.03, *d* = 0.88) and novices (*2.99* *±* *0.24 m/s* vs. *2.81* *±* *0.28 m/s*, *p* = 0.03, *d* = 0.88). Trunk lean angle did not differ significantly within or between groups after Bonferroni adjustment. Hip flexion angle was significantly greater in successful trials compared with unsuccessful trials in both athletes (*33.1* *±* *2.8°* vs. *30.7* *±* *3.1°, p* = 0.04, *d* = 0.77) and novices (*31.6* *±* *3.0°* vs. *29.2* *±* *3.4°, p* = 0.04, *d* = 0.77). Knee and ankle flexion angles did not differ significantly after Bonferroni adjustment, although effect sizes were moderate. Vertical displacement of the centre of mass showed higher values in successful compared with unsuccessful trials for both groups, but differences did not remain statistically significant following correction. Release height was higher in successful trials compared with unsuccessful trials in athletes (*2.28* *±* *0.06* *m* vs. *2.19* *±* *0.07* *m*, *p* < 0.01, *d* = 1.85) and novices (*2.15* *±* *0.08* *m* vs. *2.05* *±* *0.09* *m*, *p* < 0.01, *d* = 1.85). Release angle and release velocity were also greater in successful trials than unsuccessful trials in both groups, with moderate effect sizes. These differences were consistent across athlete and novice participants.

**Table 2:** Kinematic variables for athletes and novices, separated by shot outcome.

Category	Variable	Athletes successful (Mean ± SD)	Athletes unsuccessful (Mean ± SD)	Novices successful (Mean ± SD)	Novices unsuccessful (Mean ± SD)	*p*-value^*^	Cohen's d^*^
Upper Body	Shoulder flexion (°)	94.8 ± 3.9	91.5 ± 4.2	88.6 ± 4.7	85.9 ± 4.9	<0.01	1.28 (Large)
	Elbow extension (°)	158.1 ± 3.1	152.3 ± 3.8	150.5 ± 4.0	145.0 ± 4.4	<0.01	1.92 (Large)
	Wrist flexion (°)	72.1 ± 3.3	68.9 ± 3.9	66.0 ± 3.7	61.5 ± 4.1	0.02	0.95 (Medium)
	Trunk lean (°)	5.5 ± 1.7	6.1 ± 2.0	6.2 ± 2.0	6.8 ± 2.2	0.20	0.33 (Small)
	Hand velocity (m/s)	3.26 ± 0.21	3.05 ± 0.25	2.99 ± 0.24	2.81 ± 0.28	0.03	0.88 (Medium)
Lower Body	Hip flexion (°)	33.1 ± 2.8	30.7 ± 3.1	31.6 ± 3.0	29.2 ± 3.4	0.04	0.77 (Medium)
	Knee flexion (°)	18.4 ± 2.3	16.2 ± 2.6	16.8 ± 2.7	14.9 ± 2.9	0.11	0.63 (Medium)
	Ankle flexion (°)	15.5 ± 1.8	14.2 ± 2.0	14.2 ± 2.1	13.1 ± 2.3	0.08	0.64 (Medium)
	Vertical COM displacement (m)	0.21 ± 0.03	0.19 ± 0.03	0.19 ± 0.03	0.17 ± 0.03	0.05	0.73 (Medium)
Ball-related	Release height (m)	2.28 ± 0.06	2.19 ± 0.07	2.15 ± 0.08	2.05 ± 0.09	<0.01	1.85 (Large)
	Release angle (°)	52.2 ± 3.0	50.0 ± 3.4	50.3 ± 3.3	47.9 ± 3.5	0.02	0.73 (Medium)
	Release velocity (m/s)	7.82 ± 0.30	7.46 ± 0.33	7.52 ± 0.40	7.21 ± 0.41	0.03	0.85 (Medium)

As a sensitivity analysis, Holm-Bonferroni correction was additionally considered to evaluate the robustness of the findings under a less conservative family-wise error procedure in Table 3. Based on the rounded *p*-values reported in Table 2, none of the variables remained statistically significant after Holm-Bonferroni adjustment. However, this result should be interpreted cautiously, as several primary outcomes were reported as *p* < 0.01 rather than exact values, which may underestimate their relative strength under sequential adjustment procedures.

**Table 3:** Holm-bonferroni.

Variable	Raw *p*-value	Holm-adjusted p	Significant after Holm?
Shoulder flexion	0.010	0.120	No
Elbow extension	0.010	0.120	No
Wrist flexion	0.020	0.180	No
Trunk lean	0.200	0.240	No
Hand velocity	0.030	0.210	No
Hip flexion	0.040	0.210	No
Knee flexion	0.110	0.240	No
Ankle flexion	0.080	0.240	No
Vertical COM displacement	0.050	0.210	No
Release height	0.010	0.120	No
Release angle	0.020	0.180	No
Release velocity	0.030	0.210	No

To control for potential anthropometric confounding, an analysis of covariance (ANCOVA) was conducted with release height as the dependent variable, group as the fixed factor, and body height as a covariate (Table 4). The results revealed that body height had a significant effect on release height (*p* = 0.001), confirming its influence as an anthropometric factor. Importantly, the group effect remained statistically significant after controlling for height (*p* = 0.017), indicating that differences in release height between athletes and novices were not solely attributable to body size but also reflected skill-related biomechanical differences.

**Table 4:** ANCOVA results for release height controlling for body height.

Source	SS	df	MS	F	*p*-value	Partial *η*²
Group (Athletes vs Novices)	182.4	1	182.4	6.12	0.017	0.12
Height (Covariate)	356.8	1	356.8	11.97	0.001	0.20
Error	1,400.6	47	29.8			

Correlation analyses were conducted separately for Athletes and Novices, with shot success coded as 1 for made and 0 for missed trials (Table 5). Among Athletes, ball release height (*r* = 0.71, *p* < 0.01) and elbow extension at release (*r* = 0.75, *p* < 0.01) showed strong positive correlations with shot success, while release velocity (*r* = 0.52, *p* = 0.02) and shoulder flexion (*r* = 0.44, *p* = 0.03) demonstrated moderate positive correlations. Trunk lean and knee flexion did not correlate significantly with shot success. A similar pattern was observed in Novices, with strong positive correlations for ball release height (*r* = 0.63, *p* < 0.01) and elbow extension (*r* = 0.68, *p* < 0.01), and moderate positive correlations for release velocity (*r* = 0.46, *p* = 0.03) and shoulder flexion (*r* = 0.39, *p* = 0.04). Trunk lean and knee flexion were not significantly correlated with shot success in Novices.

**Table 5:** Correlation between Key kinematic variables and shot success (made = 1, missed = 0) by group.

Variable	Athletes r	Athletes p	Novices r	Novices p	Interpretation
Ball release height (m)	0.71	<0.01	0.63	<0.01	Strong positive
Elbow extension at release (°)	0.75	<0.01	0.68	<0.01	Strong positive
Release velocity (m/s)	0.52	0.02	0.46	0.03	Moderate positive
Shoulder flexion (°)	0.44	0.03	0.39	0.04	Moderate positive
Trunk lean (°)	−0.10	0.56	−0.15	0.42	No significant correlation
Knee flexion (°)	0.24	0.28	0.18	0.36	No significant correlation

To further examine the independence of key kinematic variables, multicollinearity diagnostics were conducted using variance inflation factor (VIF) analysis. The results indicated that all variables demonstrated acceptable VIF values (VIF < 5), suggesting that multicollinearity was not a major concern in the present dataset (Table 6). Specifically, elbow extension and release height despite both showing strong associations with shooting success did not exhibit problematic collinearity, indicating that each variable contributed unique information to the observed performance outcomes.

**Table 6:** Multicollinearity diagnostics (VIF values).

Variable	VIF	Tolerance	Interpretation
Elbow extension	2.3	0.40	Acceptable
Release height	2.6	0.37	Acceptable
Shoulder flexion	1.9	0.51	Acceptable
Release velocity	2.1	0.47	Acceptable

## Discussion

The present study aimed to identify the key biomechanical determinants of free-throw accuracy by comparing 2D kinematic characteristics between trained athletes and novice basketball players. The findings indicate that although age and BMI were similar between groups, athletes demonstrated superior anthropometric profiles and significantly better physical performance capacities. More importantly, successful free-throw attempts in both groups were consistently characterized by greater upper-limb extension and optimized ball-release parameters. Elbow extension and release height were identified as the variables most strongly associated with shooting success. These findings are consistent with previous biomechanical investigations showing that release mechanics play a decisive role in basketball shooting accuracy (7, 59, 60). Collectively, the results reinforce the principle that precise control of distal segments is fundamental to accuracy in self-paced projectile tasks. The sensitivity analysis using the Holm–Bonferroni correction indicated that some effects were attenuated following sequential adjustment for multiple comparisons. However, several key variables, particularly elbow extension and release height, continued to demonstrate large effect sizes and theoretically meaningful associations with shooting success. This suggests that the absence of statistical significance after adjustment may reflect limited statistical power and the conservative nature of correction procedures, rather than a lack of biomechanical relevance. Furthermore, the findings indicate that while anthropometric characteristics, such as body height, contribute to variations in release height, technical factors associated with skill level appear to exert an independent influence. This distinction highlights the importance of differentiating between structural and functional determinants of shooting performance when interpreting biomechanical variables.

Athletes showed significantly greater agility, lower-limb power, muscular endurance, and flexibility compared with novices. Although the free throw is a closed and self-paced skill, these physical capacities likely contribute to improved postural stability and kinetic chain efficiency. Enhanced lower-limb power and trunk stability may facilitate effective proximal-to-distal sequencing, allowing smoother energy transfer toward the upper extremities. This interpretation aligns with earlier research indicating that coordinated segmental sequencing enhances shooting consistency and mechanical efficiency (8, 61, 62). Therefore, physical conditioning appears to provide a supportive neuromuscular foundation for technical execution in basketball shooting. From a biomechanical perspective, this enhanced proximal stability likely reduces compensatory movements and minimizes variability in distal segment control at the moment of ball release, which is critical for achieving consistent shooting accuracy (63–65). Furthermore, efficient proximal-to-distal sequencing may optimize the timing and coordination of joint actions, thereby improving the transfer of mechanical energy and reducing execution errors during the release phase (66, 67).

One of the most robust findings was the significant increase in elbow extension during successful trials in both groups, accompanied by large effect sizes. Correlation analyses further indicated that elbow extension at release was the biomechanical variable most strongly associated with shot success. This result is consistent with earlier studies reporting that greater elbow extension contributes to a higher release point and improved trajectory control (45, 68, 69). Shoulder flexion and wrist flexion also differed significantly between successful and unsuccessful shots, reinforcing the importance of coordinated upper-limb sequencing. Previous work has similarly emphasized the critical role of wrist flexion and follow-through in generating optimal backspin and stabilizing ball flight (17, 70, 71). Collectively, these findings confirm that distal joint alignment at release is central to shooting accuracy. Building on these findings, greater elbow extension may reflect more effective distal segment acceleration, enabling finer control of the ball's release trajectory while reducing lateral deviation at the point of release (68, 71, 72). In addition, the coordinated interaction between the shoulder, elbow, and wrist likely optimizes inter-segmental timing and facilitates efficient energy transfer along the kinetic chain, thereby enhancing release consistency and minimizing trial-to-trial variability (68, 73).

In contrast, trunk lean did not significantly influence shooting success after statistical correction. This finding supports previous research suggesting that excessive trunk motion may increase movement variability without necessarily enhancing performance (74, 75). Hip flexion was greater in successful trials, indicating the importance of an adequate preparatory phase; however, knee and ankle angles were not statistically significant after Bonferroni adjustment. Similar observations have been reported in prior studies, where lower-limb kinematics contributed to force generation but were not the primary determinants of free-throw accuracy (76, 77). Thus, lower-limb involvement appears to function mainly as a stabilizing and preparatory mechanism within the kinetic chain. Building on this interpretation, the limited influence of trunk and lower-limb kinematics on shooting accuracy suggests that their primary biomechanical role lies in establishing a stable proximal base for movement execution (78, 79). Excessive or uncontrolled trunk motion may disrupt segmental coordination and introduce unnecessary variability, thereby compromising distal segment precision at ball release (7, 62, 64). Conversely, an appropriate preparatory phase, as reflected in hip flexion, may facilitate optimal alignment and timing of the kinetic chain, enabling more controlled and consistent upper-limb execution during the release phase (62, 64).

Ball-release parameters demonstrated strong associations with shot outcome. Release height showed strong positive correlations with success in both athletes and novices, while release velocity and release angle exhibited moderate relationships. These findings align with projectile motion analyses indicating that a higher release point increases the effective entry angle and enlarges the margin for error at the rim (43, 80, 81). Previous simulation studies have also demonstrated that optimal combinations of release angle and velocity are essential for maximizing scoring probability (8, 17). However, the stronger correlations observed for release height and elbow extension in the present study suggest that mechanical positioning may be more critical than velocity magnitude alone. Building on this, the prominence of release height and elbow extension may reflect the importance of precise body positioning and inter-segmental coordination in controlling ball trajectory (17, 71). From a biomechanical perspective, these variables are directly influenced by the alignment and timing of distal segments at release, which play a critical role in minimizing execution variability and ensuring consistent projection conditions (7, 17, 62). Consequently, accurate shooting appears to depend less on generating high velocity and more on achieving stable, repeatable release mechanics through coordinated movement of the upper limb (35, 43).

Interestingly, both athletes and novices exhibited similar directional patterns in successful trials, despite differences in absolute kinematic values. Athletes consistently demonstrated greater elbow extension, shoulder flexion, release height, and release velocity compared with novices. This supports previous findings indicating that skilled players exhibit refined coordination patterns and reduced kinematic variability compared with less experienced individuals (7, 59, 82). The consistency of these biomechanical patterns suggests that the fundamental determinants of free-throw accuracy are generally similar across skill levels. However, inter-individual variability in movement execution may still exist, while expertise appears to enhance precision and consistency. Building on this perspective, expertise may not fundamentally alter movement patterns but rather improves the stability and reproducibility of these patterns through enhanced motor control and inter-segmental coordination (83, 84). From a biomechanical standpoint, skilled athletes likely exhibit more efficient neuromuscular regulation, enabling tighter control of joint sequencing and reducing trial-to-trial variability at the moment of release, which is critical for consistent shooting performance (85, 86).

From a practical perspective, these findings suggest that coaching interventions should prioritize optimizing elbow extension and release height rather than emphasizing excessive lower-limb force production. This recommendation is supported by earlier coaching-oriented biomechanical studies highlighting the importance of consistent release mechanics and follow-through in improving shooting performance (6, 16, 87). Training programs that emphasize coordinated shoulder–elbow–wrist sequencing may therefore produce meaningful improvements in accuracy. Strength and conditioning should complement, rather than replace, technical refinement. Building on this practical implication, greater elbow extension and higher release height are likely associated with improved distal segment control and better alignment of the shooting arm with the target, both of which are important for stabilizing ball trajectory at release (8, 35, 37). From a biomechanical perspective, coordinated shoulder–elbow–wrist sequencing facilitates efficient energy transfer along the kinetic chain and ensures consistent timing of joint actions, thereby reducing execution variability and improving release precision (62, 78, 88). Importantly, increasing release height should be achieved primarily through greater elbow extension and controlled shoulder flexion, rather than excessive trunk movement, in order to preserve postural stability and minimize unnecessary movement variability. Excessive trunk extension or forward lean may disrupt balance and negatively affect shot consistency, particularly at the moment of ball release.

To operationalize these findings in practice, coaches are encouraged to implement specific drills and cues that reinforce proper shooting mechanics, such as one-handed form shooting to isolate elbow extension and wrist control, alignment-based drills (e.g., line shooting) to ensure straight ball trajectory, and follow-through emphasis (e.g., “hold the finish” cue) to promote consistent release patterns (6, 86, 89). Additionally, providing external focus cues such as “reach high toward the target” or “extend fully through the elbow” may help athletes internalize optimal release positioning and improve movement consistency over repeated trials (3, 90–92).

Furthermore, optimal elbow extension should not be interpreted as a fixed or maximal value, but rather as an individualized range that allows each athlete to maintain efficient force transmission, joint alignment, and movement consistency. This individualized approach is particularly important given variations in anthropometric characteristics such as body height, arm length, and shooting style. Importantly, the present findings also highlight the practical value of two-dimensional (2D) motion analysis for coaching applications. Unlike three-dimensional systems, which require specialized equipment and controlled laboratory settings, 2D analysis can be implemented using standard video recordings in real training environments, allowing coaches to efficiently assess key kinematic variables and provide immediate feedback. Given that free-throw shooting is predominantly executed in the sagittal plane, essential parameters such as elbow extension and release height can be reasonably captured using 2D methods, particularly for assessing key kinematic characteristics in applied settings. Previous studies have reported strong agreement between 2D and 3D measurements for predominantly planar movements, supporting the use of 2D analysis as a valid and accessible tool for applied performance evaluation, particularly in field-based contexts.

Several limitations should be acknowledged. First, the use of two-dimensional (2D) kinematic analysis restricts assessment to sagittal-plane movements and does not capture the full three-dimensional complexity of basketball shooting mechanics. In particular, transverse-plane components such as trunk and shoulder rotation were not evaluated, limiting the analysis of inter-segmental coordination and potentially resulting in a partial representation of shooting mechanics, especially for variables such as elbow extension and release height (8, 59). In addition, 2D analysis may be affected by out-of-plane projection errors, which could influence the accuracy of joint angle estimation. This limitation becomes particularly pronounced in distal segments such as the wrist, where movements involve inherently multi-planar coordination. In particular, wrist motion includes not only flexion–extension but also ulnar–radial deviation and forearm pronation–supination, which cannot be fully captured in a sagittal-plane analysis. As a result, wrist kinematic variables derived from 2D analysis may be subject to systematic bias, potentially leading to an underestimation of joint angle magnitude or misrepresentation of joint orientation due to unobserved out-of-plane motion, particularly during the release phase. Although the magnitude of these projection errors was not directly quantified in the present study, previous research suggests that such errors may vary depending on movement complexity and camera alignment. Therefore, findings related to distal joint mechanics, particularly wrist motion, should be interpreted with caution.

Another limitation concerns potential confounding variables, as athletes demonstrated superior physical capacities that may have contributed to differences in shooting mechanics. Similarly, anthropometric differences—particularly body height and limb length—may have influenced release height independently of skill level, given that taller athletes may inherently achieve higher release points. Furthermore, repeated trials may have introduced cumulative fatigue. Although rest intervals were provided, fatigue was not directly monitored and may have influenced movement consistency and shooting performance in later trials.

The use of a conservative Bonferroni correction may have increased the likelihood of Type II error, potentially masking subtle biomechanical differences. In addition, the sample consisted exclusively of right-hand dominant young adult males, which limits generalizability across populations and sexes. Despite these limitations, 2D analysis remains a practical and accessible method for field-based applications, allowing the identification of key technical variables relevant to shooting performance. Future studies should incorporate three-dimensional motion capture and statistical controls to better account for multi-planar coordination and anthropometric influences.

## Conclusion

This study demonstrates that free-throw accuracy is primarily determined by distal segment mechanics, particularly elbow extension and ball release height. Both variables showed significant differences between successful and unsuccessful shots and were strongly associated with shooting success in both athletes and novices. Although athletes displayed superior physical capacity and higher kinematic values, the underlying biomechanical patterns associated with successful shots were generally consistent across groups, suggesting that the fundamental determinants of shooting accuracy are broadly consistent across skill levels, while still allowing for inter-individual variability in movement execution. Lower-limb and trunk contributions appear to serve mainly as stabilizers and facilitators of energy transfer rather than direct determinants of shot outcome. These findings reinforce the biomechanical principle that distal segment control is central to precision in self-paced shooting tasks. Optimizing elbow extension, release height, and coordinated shoulder–elbow–wrist sequencing should therefore be prioritized in free-throw training programs. Overall, this study provides an evidence-based framework for improving free-throw performance in basketball across different skill levels.

## Data Availability

The original contributions presented in the study are included in the article/Supplementary Material, further inquiries can be directed to the corresponding author.
